# Supplementary material for: Non-linear Terahertz driving of plasma waves in layered cuprates
Source: Nat Commun. 2021 Feb 2;12:752. doi: 10.1038/s41467-021-21041-6 (PMC7854596; doi:10.1038/s41467-021-21041-6)
Supplement: Supplementary file 1 — Supplementary Information [file 41467_2021_21041_MOESM1_ESM.pdf]

Non-linear Terahertz driving of plasma waves in layered cuprates

Francesco Gabriele,[1] Mattia Udina,[1] and Lara Benfatto[1]

[1] *Department of Physics and ISC-CNR, "Sapienza" University of Rome, P.le A. Moro 5, 00185 Rome, Italy*

Supplementary Note 1: Analogy with the non-linear sine-Gordon equations for the JPM

As discussed in the Method section, to obtain the effective action for the phase degrees of freedom we rely on the microscopic derivation described e.g. in Ref.s[1–3]. The phase-only model is obtained from a microscopic SC model, and the plasma mode emerges after integrating out density fluctuations, mediated by the Coulomb potential. An alternative but equivalent method is instead the one followed e.g. in Ref.s[4–8], where one deals with the equations of motion for the plasmon, coupled to the electromagnetic fields. The connection between the two approaches has been derived in details in Ref.s[4,5]. Once more, the authors start from a microscopic SC layered model, and integrate out the fermionic degrees of freedom in order to build up an effective action for the phase field. The effective quantum action then reads:

$$S = \sum_{n,\mathbf{r}_i} \int d\tau \left\{ \frac{C_0}{2} \left(\frac{1}{2e} \frac{\partial \phi_n}{\partial \tau} \right)^2 - J_\perp \cos(\phi_{n,\mathbf{r}_i}(\tau) - \phi_{n+1,\mathbf{r}_i}(\tau)) - \sum_{\alpha=x,y} J_\parallel \cos(\phi_{n,\mathbf{r}_i}(\tau) - \phi_{n,\mathbf{r}_i+\hat{\delta}_\alpha}(\tau)) \right\}, \quad (1)$$

where $\mathbf{r}_i$ is the two-dimensional in-plane coordinate running over each SC layer and δ_α is the in-plane versor along the direction $\alpha = x, y$. In Eq. (1) the quantum term accounts for the capacitive coupling $C_0 = s/4\pi R_D^2$ between the planes, s and R_D being, respectively, the layer thickness and the Debye length. By retaining leading orders in ϕ in the cosine terms, the Gaussian action from Eq. (1) describes a sound mode, in full analogy with Eq. (15) of the main text in the absence of RPA resummation of the density response. Indeed, as emphasized in the manuscript, the presence of long-range interactions is crucial in order to lift the sound mode to a plasmon. In Ref.s[4,5] this is achieved by adding explicitly the electric and magnetic fields, and the corresponding scalar and vector potentials. To describe the out-of-plane JPM one needs an electric field $E^z_{n,n+1}$ polarized perpendicularly to the planes. The magnetic field will then lie in the plane, and we can take without loss of generality $B^y_{n,n+1}$ along the y in-plane direction. Hence the Gaussian action becomes:

$$S = \sum_{n,\mathbf{r}_i} \int d\tau \left\{ \frac{C_0}{2} \left(\frac{\partial \tilde{\phi}_n}{\partial \tau} + V_n \right)^2 + \frac{\tilde{J}_\perp}{2} (\tilde{\phi}_n - \tilde{\phi}_{n+1})^2 + \frac{\tilde{J}_\parallel}{2} \left(\Delta_x \tilde{\phi}_n - \frac{1}{c} A^x_n \right)^2 + \frac{C}{2} \left[(E^z_{n,n+1})^2 + \frac{1}{\varepsilon} (B^y_{n,n+1})^2 \right] \right\}, \quad (2)$$

where $\Delta_x \phi_n \equiv \phi_{n,\mathbf{r}_i}(\tau) - \phi_{n,\mathbf{r}_i+\hat{x}}$, a and $D = d + s \simeq d$ are, respectively, the in-plane and out-of-plane lattice spacings and $C = \frac{\varepsilon}{4\pi D}$. We also introduced the rescaled quantities $\tilde{\phi} \equiv \frac{\phi_0}{2\pi c} \phi$ and $\tilde{J} \equiv \left(\frac{2\pi c}{\phi_0} \right)^2 J$. By means of the Maxwell equations one can replace $E^z_{n,n+1} = (V_n - V_{n+1})/D$ and $B^y_{n,n+1} = (A^x_{n+1} - A^x_n)/D$ into Eq. (2). The explicit integration of the e.m. potentials then leads to:

$$S^G_\perp = \frac{C}{2} \sum_{i\omega_m,\mathbf{q}} \frac{4\sin^2(k_z d/2)}{1 + 4\alpha \sin^2(k_z d/2)} \left[\omega_m^2 + \omega_P^2(\mathbf{q}) \right] |\phi(i\omega_m, \mathbf{q})|^2, \quad (3)$$

where $C = \varepsilon/(4\pi D)$, $\alpha = C/C_0$ and

$$\frac{\omega_P^2(\mathbf{q})}{\omega_J^2} = 1 + 4\alpha \sin^2(k_z d/2) + \frac{4 (\lambda_c/\xi_0)^2 \sin^2(k\xi_0/2)}{1 + 4(\lambda_{ab}/d)^2 \sin^2(k_z d/2)} \quad (4)$$

describes the full dispersion of the plasma mode as a function of $\mathbf{q} = (k_z, k)$, with k laying in the in-plane propagation direction. Here ξ_0 is the SC coherence length, which sets the in-plane lattice spacing, and $\lambda_{ab(c)}$ is the in-plane (out-of-plane) penetration depth. The pole equation for the Gaussian phase mode, i.e. $\omega^2 = \omega_P^2(\mathbf{q})$, is completely equivalent to the solution of the linearized sine-Gordon equation for Josephson plasma waves previously addressed in the literature[4–8]. In cuprates the constant α is usually very small, so the main dispersion of the plasmon comes from the last term of Eq. (4), which accounts for the inductive coupling between planes. In this approximation, the

Gaussian phase fluctuations identify a collective mode whose energy dispersion is obtained as the pole of the Guassian propagator for phase fluctuations:

$$\langle|\phi(i\omega_m,\mathbf{q})|^2\rangle = \frac{1}{4\sin^2(k_z d/2)\left[\omega_m^2+\omega_P^2(\mathbf{q})\right]}. \quad (5)$$

By analytical continuation $i\omega_m \to \omega + i\delta$ in Eq. (5) we then get

$$\omega^2 = \omega_P^2(\mathbf{q}) = \omega_J^2\left[1+\frac{4\left(\lambda_c/\xi_0\right)^2\sin^2\left(k\xi_0/2\right)}{1+(\lambda_{ab}/d)^2 4\sin^2(k_z d/2)}\right]. \quad (6)$$

The relation (6) is the same that one obtains by using the equation of motion approach discussed in Ref.s[4–8]. In this case, one introduces directly the variable $\theta_n \equiv \phi_n - \phi_{n+1}$ which represents the phase difference between nearest-neighbour layers. It is then shown to satisfy the equation of motion[6]

$$\left(1-\frac{\lambda_{ab}^2}{d^2}\partial_n^2\right)\left[\frac{1}{\omega_J^2}\frac{\partial^2\theta_n}{\partial t^2}+\sin(\theta_n)\right]-\frac{\lambda_c^2}{\xi_0^2}\partial_x^2\theta_n = 0, \quad (7)$$

where $\partial_n^2 f_n \equiv f_{n+1}+f_{n-1}-2f_n$ is the second-order discrete differential operator along the z direction, and analogously ∂_x^2 for the x direction. As one can easily check, when $\sin\theta_n \approx \theta_n$ Eq. (7) admits a wave solution $\theta_n(x=m\xi_0,t) \propto \exp[i(kx+k_z nd-\omega t)]$ where the frequency ω and the momentum $\mathbf{q}=(k,k_z)$ satisfy the Eq. (6). In the approach of Ref.s[4–8], based on the study of the equation of motions, the electromagnetic field is completely eliminated and the non-linear effects are included by retaining the full $\sin\theta_n$ term in the sine-Gordon model (7). In this approach, a real $\mathbf{q}$ solution for a propagating waves is only possible if one retains the full momentum dispersion in Eq. (4). In contrast, in our approach the plasma mode is first computed at Gaussian level, and then non-linear effects originate by retaining in Eq. (2) the full cosine term in Eq. (1), which is responsible for the non-linear coupling to the gauge potential. The phase mode is then integrated out in order to obtain the complete electromagnetic response, as required to describe non-linear effects in the currents, see Eq. (3) and (6) in the manuscript. This is indeed the same approach that has been used so far to investigate the nature and the non-linear response of the SC Higgs mode, by means of two-dimensional models able to describe the in-plane response[9]. In this view, the dispersion of the plasma mode (as well as the Higgs mode in the case of amplitude fluctuations) is quantitatively irrelevant, and what matters is only the resonance process which occurs when the pumping frequency matches the value of the Josephson frequency ω_J. For this reason we retained in our calculation the $\mathbf{q}\to 0$ long-wavelength limit in the phase propagator (5), as done in Eq. (4) of the manuscript.

To account also for possible dissipative effects one usually adds in the equation (7) also a term linear in the time derivative:

$$\left(1-\frac{\lambda_{ab}^2}{d^2}\partial_n^2\right)\left[\frac{1}{\omega_J^2}\frac{\partial^2\theta_n}{\partial t^2}+r\frac{\partial\theta_n}{\partial t}+\sin(\theta_n)\right]-\frac{\lambda_c^2}{\xi_0^2}\partial_x^2\theta_n = 0. \quad (8)$$

This additional tern can be justified once more at microscopic level by following the derivation of Ref.[2]. Indeed, the $D_s\mathbf{q}^2$ term in Eq. (15) of the main text originates from the long-wavelength limit of the current-current correlation function. Taking into account also the presence of a regular part σ_{reg} of the low-frequency conductivity due to normal quasiparticles, one can easily show[2] that Eq. (4) of the main text gets modified as:

$$S_\perp^{G,diss} = \frac{v}{2d^2}\sum_{i\omega_m,k_z} 4\sin^2(k_z d/2)\left(\omega_m^2+\omega_J^2+\sigma_{reg}\mid\omega_m\mid\right)\mid\phi(i\omega_m,k_z)\mid^2. \quad (9)$$

Using again the analogy between the pole of the phase propagator (5) and the solution of the equations of motion (8) one understands why absorption by normal quasiparticles leads to dissipation of the plasma waves.

Supplementary Note 2: Derivation of the non-linear optical kernel

We first focus on the out-of-plane THG. As shown in Eq. (5) of the main text, by expanding the first cosine in Eq. (1) (once the minimal-coupling $\phi_n - \phi_{n+1} \to \phi_n - \phi_{n+1} - \frac{2\pi}{\Phi_0}dA_z$ has been performed) we find that:

$$S = S_\perp^G - \sqrt{\frac{T}{N_s}}\frac{\pi^2 d^2}{\Phi_0^2}\sum_{n,\mathbf{r}_i} J_\perp\int d\tau A_z^2(\tau)\left(\phi_{n,\mathbf{r}_i}(\tau)-\phi_{n+1,\mathbf{r}_i}(\tau)\right)^2+\ldots=$$

$$= S_\perp^G - \sqrt{\frac{T}{N_s}}\frac{\pi^2 d^2}{\Phi_0^2}\sum_{i\omega_m,i\omega_m'}\sum_{k_z} J_\perp k_z^2 A_z^2(i\omega_m - i\omega_m')\overline{\phi}(i\omega_m,k_z)\phi(i\omega_m',k_z)+\ldots \quad (10)$$

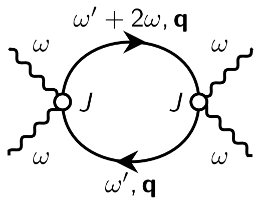

Supplementary Figure 1. Feynman diagram representing the fourth-order term (16). Here wavy/solid lines represent the vector potential/phase mode, respectively. The phase mode is computed at Gaussian level and it is given by Eq. (5).

where dots denote additional terms not relevant for the $\chi^{(3)}$ response, and we used the $k_z \to 0$ limit for the finite difference along z. Starting from Eq. (10) we can easily obtain the out-of-plane non-linear optical kernel. After adopting the notation $q \equiv (i\omega_m, k_z)$ the partition function of the system can be rewritten as:

$$Z = \int \mathcal{D}[\phi, \overline{\phi}] e^{-\frac{1}{2}\sum_{q,q'}\left[D(q,q')+R(q,q')\right]\overline{\theta}(q)\theta(q')}, \tag{11}$$

where:

$$D(q, q') = v\left(\omega_m^2 + \omega_J^2\right) k_z^2 \delta(i\omega_m - i\omega'_m)\delta(k_z - k'_z) \tag{12}$$

$$R(q, q') = -\sqrt{\frac{T}{N_s}} \frac{2\pi^2 d^2}{\Phi_0^2} J_\perp k_z^2 A_z^2(i\omega_m - i\omega'_m)\delta(k_z - k'_z). \tag{13}$$

At this point we integrate out the phase fluctuations: this gives a contribution beyond RPA approximation to the effective action equivalent to one-loop corrections in the phase mode. With straightforward algebra we obtain:

$$S^{(eff)} = \sum_{n=1}^{+\infty} \frac{\mathrm{Tr}_q\{(D^{-1}R)^n\}}{n}. \tag{14}$$

Therefore, the fourth-order action in $\mathbf{A}$ $S^{(4)}$ is:

$$S_\perp^{(4)} = K_0 J_\perp^2 \sum_{i\omega_m} A_z^2(i\omega_m) T \sum_{i\omega'_m} \frac{1}{[(\omega_m + \omega'_m)^2 + \omega_J^2][(\omega'_m)^2 + \omega_J^2]} A_z^2(-i\omega_m) \tag{15}$$

where we retained the only term contributing to the THG and we put all the multiplicative constants into K_0. Eq. (15) corresponds to the Feynman diagram of Fig. 1b of the main text, that we report in Fig. 1 with the explicit frequency dependence. Here each solid line denote a phase propagator, given by Eq. (5) above. Notice that since the gauge field couples to the phase gradient along z, see Eq. (13), each k_z^2 term from the vertex of the diagram in Fig. 1 compensates a $1/k_z^2$ term from the denominator of the phase propagator in Eq. (5).

After computing the Matsubara sum, i.e. $T\sum_{i\omega'_m} \frac{1}{(\omega_m+\omega'_m)^2+\omega_J^2} \frac{1}{\omega_m'^2+\omega_J^2} = \frac{\coth\left(\frac{\beta\omega_J}{2}\right)}{\omega_J(\omega_m^2+4\omega_J^2)}$, the fourth-order effective action becomes:

$$S^{(4)} = \sum_{i\omega_m} A_z^2(i\omega_m) K_\perp(i\omega_m) A_z^2(-i\omega_m), \tag{16}$$

where

$$K_\perp(i\omega_m) = K_0 \frac{J_\perp^2}{\omega_J} \frac{\coth(\beta\omega_J/2)}{4\omega_J^2 + \omega_m^2} \tag{17}$$

is the out-of-plane non-linear optical kernel.
We consider now the case of an in-plane polarized external field. Following the same scheme adopted for the out-of-plane case we find that the in-plane fourth-order effective action is:

$$S^{(4)} = \sum_{i\omega_m} \sum_{i,j} A_i^2(i\omega_m) K_{ij}(i\omega_m) A_j^2(-i\omega_m), \tag{18}$$

where $K_{ij}(i\omega_m) = M_{ij}\frac{J_\parallel^2}{\omega_J^\parallel}\frac{\coth(\beta\omega_J^\parallel/2)}{4(\omega_J^\parallel)^2+\omega_m^2}$. $M_{ij} = K_0\begin{pmatrix}\sum_{k_x,k_y}\frac{k_x^4}{\mathbf{k}^4} & \sum_{k_x,k_y}\frac{k_x^2k_y^2}{\mathbf{k}^4}\\ \sum_{k_x,k_y}\frac{k_x^2k_y^2}{\mathbf{k}^4} & \sum_{k_x,k_y}\frac{k_x^4}{\mathbf{k}^4}\end{pmatrix}$ is the polarization-dependent tensor, whose components read:

$$M_{xx} = M_{yy} \simeq \int_0^{2\pi} d\phi\cos^4\phi = \frac{3\pi}{4} \tag{19}$$

$$M_{xy} = M_{yx} \simeq \int_0^{2\pi} d\phi\cos^2\phi\sin^2\phi = \frac{\pi}{4}. \tag{20}$$

Hence the tensor components of the in-plane non-linear optical kernel are those enlisted in Eq. (12) of the main text.

Finally, to add the effects of dissipation we can add the additional $\sigma_{reg}|\omega_m|$ term of Eq. (9). In this case, the calculation can be done by introducing the spectral function of the phase mode $A(z) \equiv \frac{z\sigma}{\left(z^2-\omega_J^2\right)^2+z^2\sigma_{reg}^2}$. We then find that, in general, the linear kernel reads:

$$K^{(diss)}(i\omega_m) = K_0J_\perp^2\int_{-\infty}^{+\infty}\frac{dz}{\pi}\int_{-\infty}^{+\infty}\frac{dz'}{\pi}A(z)A(z')\frac{b(z)-b(z')}{z'-z+i\omega_m}, \tag{21}$$

where $b(z) = \frac{1}{e^{\beta z}-1}$ is the Bose function. If $\sigma << \omega_J$ one can show that Eq. (21) can be approximated, after analytical continuation, as:

$$K^{(diss)}(\omega) \simeq K_0\frac{J_\perp^2}{\omega_J}\frac{\coth\left(\frac{\beta\omega_J}{2}\right)}{4\omega_J^2-\left(\omega+i\sigma_{reg}\right)^2}. \tag{22}$$

Eq. (22) is the formula used, indeed, to compute all the quantities of interest in the main text. In analogy with Ref.[6] we also assumed that

$$\sigma_{reg} = \gamma(T) \equiv \gamma_0 + r(T) \tag{23}$$

where γ_0 is a small regularization constant, which prevents the non-linear optical kernel $K^{(diss)}$ to be ill-defined at $T = 0$. Both γ_0 and r_0 are fixed by considering the number of oscillations observed experimentally in the pump-probe set up of Ref.[7] at low temperatures.

Supplementary Note 3: Persistence of the in-plane superfluid stiffness above T_c

As discussed in the main text, the experimentally observed THG above the critical temperature in cuprate superconductors is intimately linked to the persistence of the superfluid density $J_\Omega(T)$ for $T > T_c$, where we added the Ω subscript to highlight the fact that what matters is the superfluid stiffness measured at a finite frequency Ω. This is determined experimentally as $J_\Omega(T) = \sigma_2(\Omega,T)/\Omega$, and it can differ in general from the real phase rigidity, defined as its $\Omega \to 0$ limit, i.e. $J(T) = \lim_{\Omega\to 0}\sigma_2(\Omega,T)/\Omega$. Here $\sigma_2(\Omega,T)$ denotes the imaginary part of the optical conductivity measured at the THz frequency Ω at which the THG experiment is carried out. In any superconductor, both in conventional ones (see e.g. Ref.[10]) and in cuprates (see e.g. Ref.s[11–13]), $J(T)$ vanishes exactly at T_c, but the phase rigidity measured at the finite length scale set up by the finite frequency Ω of the experiment survives in general in a certain range of temperatures above T_c. This finding can be ascribed both to SC fluctuations above T_c and to intrinsic inhomogeneity of the SC properties, with a possible cooperative effect of both phenomena. In the case of Gaussian (amplitude and phase) fluctuations the intrinsic mechanism relies on the fact that preformed Cooper pairs appear bounded already above T_c at the finite length scale set by the probe frequency, leading to a superfluid response[14]. The range of temperature above T_c where this effect is appreciable depends on several factors: even in a conventional BCS superconductor as NbN, Ref.[10] has shown an enhancement of the fluctuation regime as the system approaches the superconductor-to-insulator transition, where it gets intrinsically inhomogeneous. A second mechanism possibly responsible for the survival of $J_\Omega(T)$ above T_c can be linked to phase fluctuations only, eventually of the Berezinskii-Kosterlitz-Thoulee type, which are expected to be enhanced in cuprates due to the quasi-two-dimensional nature of the pairing[11]. Also in this case, the existence of an inhomogeneous superconducting ground state strongly enhances the effect of fluctuations, leading to pronounced tails above T_c[15,16]. While in conventional superconductors

some semi-quantitative work has been done to capture these phenomena[10,15,16], in cuprates a full microscopic understanding of this issue is still nowadays an open problem, whose explanation goes beyond the scope of our manuscript. Nonetheless, since J_Ω sets the energy scale for the THG, as soon as J_Ω persists above T_c also the THG signal remains finite.

For the matter of a qualitative comparison with the experiments, we then decided to simulate the survival of J_Ω above T_c by simply implementing the effects of the inhomogeneous SC background, whose existence has been proven by STM measurements in cuprates[17,18]. We then modelled the persistence of the in-plane superfluid stiffness above the critical temperature by assuming phenomenologically an inhomogeneous ground state. We then model the system with a distribution of local T_c^i values Gaussian distributed around a mean value T_c, which represents the temperature where the superfluid stiffness vanishes in the $\Omega \to 0$ limit. The global $J_\Omega(T)$ stiffness is then obtained as the average of the local $J^i(T)$ values. The existence of regions with local T_c^i larger than the average one produces a tail above T_c. The range of temperature where the stiffness survives depends in turn on the width of the Gaussian distribution of the local critical temperatures. This is shown in Fig. 2, where we show results for two Gaussian probability densities (orange and blue filled curves), with standard deviations $\sigma = 0.2$ and $\sigma = 0.5$, respectively. The $\sigma = 0.5$ curve has been used in Fig. 3c of the main paper to simulate the resulting THG. We stress once more that for what concerns the THG the exact mechanism underlying the survival of J_Ω above T_c is not relevant. Indeed, for pump frequencies much smaller than $\omega_{J,0}^{\parallel}$ what matters is just the overall temperature scaling of the stiffness prefactor in Eq. (11) of the manuscript, that sets the scale of the THG temperature variation.

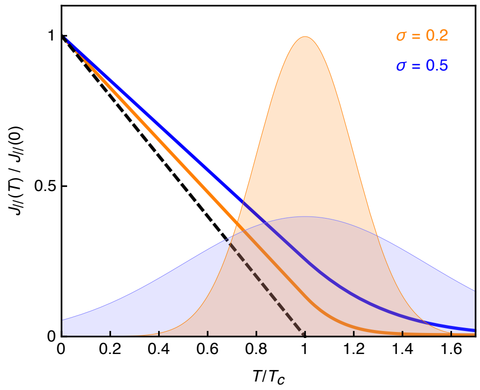

Supplementary Figure 2. Normalized in-plane superfluid stiffness as a function of temperature. The dashed black line shows the usual linear behavior with a vanishing stiffness at T_c in the $\Omega \to 0$ limit. The orange and blue plain lines show the average stiffness emerging from a random distribution of local T_c^i values, distributed according to the Gaussian probability densities shown in orange ($\sigma = 0.2$) and blue ($\sigma = 0.5$).

[1] N. Nagaosa and S. Heusler, *Quantum Field Theory in Condensed Matter Physics*, Texts and monographs in physics (Springer, 1999).

[2] L. Benfatto, S. Caprara, C. Castellani, A. Paramekanti, and M. Randeria, "Phase fluctuations, dissipation, and superfluid stiffness in d-wave superconductors," Phys. Rev. B **63**, 174513 (2001).

[3] L. Benfatto, A. Toschi, and S. Caprara, "Low-energy phase-only action in a superconductor: A comparison with the XY model," Phys. Rev. B **69**, 184510 (2004).

[4] M. Machida, T. Koyama, and M. Tachiki, "Dynamical breaking of charge neutrality in intrinsic josephson junctions: Common origin for microwave resonant absorptions and multiple-branch structures in the $I - V$ characteristics," Phys. Rev. Lett. **83**, 4618–4621 (1999).

[5] M. Machida, T. Koyama, A. Tanaka, and M. Tachiki, "Theory of the superconducting phase and charge dynamics in intrinsic josephson-junction systems: microscopic foundation for longitudinal josephson plasma and phenomenological dynamical equations," Physica C: Superconductivity **331**, 85 – 96 (2000).

[6] Sergey Savel'ev, V A Yampol'skii, A L Rakhmanov, and Franco Nori, "Terahertz josephson plasma waves in layered superconductors: spectrum, generation, nonlinear and quantum phenomena," Reports on Progress in Physics **73**, 026501 (2010).

[7] S. Rajasekaran, E. Casandruc, Y. Laplace, D. Nicoletti, G. D. Gu, S. R. Clark, D. Jaksch, and A. Cavalleri, "Parametric amplification of a superconducting plasma wave," Nature Physics **12**, 1012 (2016).

[8] S. Rajasekaran, J. Okamoto, L. Mathey, M. Fechner, V. Thampy, G. D. Gu, and A. Cavalleri, "Probing optically silent superfluid stripes in cuprates," Science **359**, 575–579 (2018).

[9] T. Cea, C. Castellani, and L. Benfatto, "Nonlinear optical effects and third-harmonic generation in superconductors: Cooper pairs versus higgs mode contribution," Phys. Rev. B **93**, 180507 (2016).

[10] Mintu Mondal, Anand Kamlapure, Somesh Chandra Ganguli, John Jesudasan, Vivas Bagwe, Lara Benfatto, and Pratap Raychaudhuri, "Enhancement of the finite-frequency superfluid response in the pseudogap regime of strongly disordered superconducting films," Scientific Reports **3**, 1357 (2013).

[11] J. Corson, R. Mallozzi, J. Orenstein, J. N. Eckstein, and I. Bozovic, "Vanishing of phase coherence in underdoped $Bi_2Sr_2CaCu_2O_{8+\delta}$," Nature **398**, 221–223 (1999).

[12] L. S. Bilbro, R. Valdés Aguilar, G. Logvenov, O. Pelleg, I. Božović, and N. P. Armitage, "Temporal correlations of superconductivity above the transition temperature in $La_{2-x}Sr_xCuO_4$ probed by terahertz spectroscopy," Nature Physics **7**, 298–302 (2011).

[13] Kota Katsumi, Zhi Zhong Li, Hélène Raffy, Yann Gallais, and Ryo Shimano, "Superconducting fluctuations probed by the higgs mode in $Bi_2Sr_2CaCu_2O_{8+x}$ thin films," Phys. Rev. B **102**, 054510 (2020).

[14] Anatoly Larkin and Andrei Varlamov, *Theory of fluctuations in superconductors*, revised ed. ed., International series of monographs on physics, 127 (Oxford University Press, 2005).

[15] Rini Ganguly, Dipanjan Chaudhuri, Pratap Raychaudhuri, and Lara Benfatto, "Slowing down of vortex motion at the berezinskii-kosterlitz-thouless transition in ultrathin NbN films," Phys. Rev. B **91**, 054514 (2015).

[16] G. Venditti, J. Biscaras, S. Hurand, N. Bergeal, J. Lesueur, A. Dogra, R. C. Budhani, Mintu Mondal, John Jesudasan, Pratap Raychaudhuri, S. Caprara, and L. Benfatto, "Nonlinear $I-V$ characteristics of two-dimensional superconductors: Berezinskii-kosterlitz-thouless physics versus inhomogeneity," Phys. Rev. B **100**, 064506 (2019).

[17] Kenjiro K. Gomes, Abhay N. Pasupathy, Aakash Pushp, Shimpei Ono, Yoichi Ando, and Ali Yazdani, "Visualizing pair formation on the atomic scale in the high-tc superconductor $Bi_2Sr_2CaCu_2O_{8+\delta}$," Nature **447**, 569 (2007).

[18] J. W. Alldredge, Jinho Lee, K. McElroy, M. Wang, K. Fujita, Y. Kohsaka, C. Taylor, H. Eisaki, S. Uchida, P. J. Hirschfeld, and J. C. Davis, "Evolution of the electronic excitation spectrum with strongly diminishing hole density in superconducting $Bi_2Sr_2CaCu_2O_{8+\delta}$," Nat. Phys. **4**, 319 (2008).
